# Phenylpropanoid Metabolism in *Phaseolus vulgaris* during Growth under Severe Drought

**DOI:** 10.3390/metabo14060319

**Published:** 2024-05-31

**Authors:** Luis Eduardo Peña Barrena, Lili Mats, Hugh J. Earl, Gale G. Bozzo

**Affiliations:** 1 Department of Plant Agriculture, University of Guelph, 50 Stone Road East, Guelph, ON N1G 2W1, Canada; lpenabar@uoguelph.ca (L.E.P.B.); hjearl@uoguelph.ca (H.J.E.); 2 Guelph Research and Development Centre, Agriculture and Agri-Food Canada, 93 Stone Road West, Guelph, ON N1G 5C9, Canada; lili.mats@canada.ca

**Keywords:** drought, flavonol glycosides, isoflavones, *Phaseolus vulgaris*, phenylpropanoids

## Abstract

Drought limits the growth and development of *Phaseolus vulgaris* L. (known as common bean). Common bean plants contain various phenylpropanoids, but it is not known whether the levels of these metabolites are altered by drought. Here, BT6 and BT44, two white bean recombinant inbred lines (RILs), were cultivated under severe drought. Their respective growth and phenylpropanoid profiles were compared to those of well-irrigated plants. Both RILs accumulated much less biomass in their vegetative parts with severe drought, which was associated with more phaseollin and phaseollinisoflavan in their roots relative to well-irrigated plants. A sustained accumulation of coumestrol was evident in BT44 roots with drought. Transient alterations in the leaf profiles of various phenolic acids occurred in drought-stressed BT6 and BT44 plants, including the respective accumulation of two separate caftaric acid isomers and coutaric acid (isomer 1) relative to well-irrigated plants. A sustained rise in fertaric acid was observed in BT44 with drought stress, whereas the greater amount relative to well-watered plants was transient in BT6. Apart from kaempferol diglucoside (isomer 2), the concentrations of most leaf flavonol glycosides were not altered with drought. Overall, fine tuning of leaf and root phenylpropanoid profiles occurs in white bean plants subjected to severe drought.

## 1. Introduction

Drought affects most plants cultivated on arable land worldwide. A rise in atmospheric CO_2_ and/or temperature is expected to exacerbate the incidence and severity of drought in some parts of the globe within this century [1,2]. *Phaseolus vulgaris* L. (common bean; also known as dry bean) produces seeds that are rich in protein [2]. In 2022, nearly 37 million hectares of common beans were harvested worldwide [3]. Some common bean production is in semi-arid regions of the western hemisphere that are prone to drought [4]. Like other legumes, a reduction in common bean yield is proportional to the percent reduction in soil moisture [5]. Bean yield losses due to drought can represent 60–70% of all production [5,6,7]. In Canada, *P. vulgaris* is produced within the prairie provinces and southern Ontario. Drought events are expected to be more frequent in the southern prairies with incremental warming and a consequent rise in evaporation [8]. Transient drought events are typical through the summer months, where most agriculture is produced in southern Ontario, and major drought events have occurred in this region within the last century [9]. 

In most plants, a canonical response to soil water deficit is the transport of abscisic acid (ABA) from roots to shoots, as well as de novo biosynthesis of ABA in leaves and their guard cells [10]. ABA induces the buildup of reactive oxygen species (ROS) in leaf guard cells, leading to the closure of stomata [11]. ROS, such as superoxide and hydrogen peroxide, are amassed in drought-stressed plants [12]. Too much ROS in plants can lead to cellular damage and tissue death, and advanced senescence [13]. ROS homeostasis within plants depends on their dissipation by both enzymatic (e.g., superoxide dismutase, catalase, ascorbate peroxidase) and non-enzymatic (e.g., ascorbate, flavonoids) ROS scavenging mechanisms [13]. Tomato plants that hyperaccumulate flavonoids undergo less ROS-related stomatal closure [11], but it is unclear how this relates to drought tolerance in plants. 

**Figure 1:**
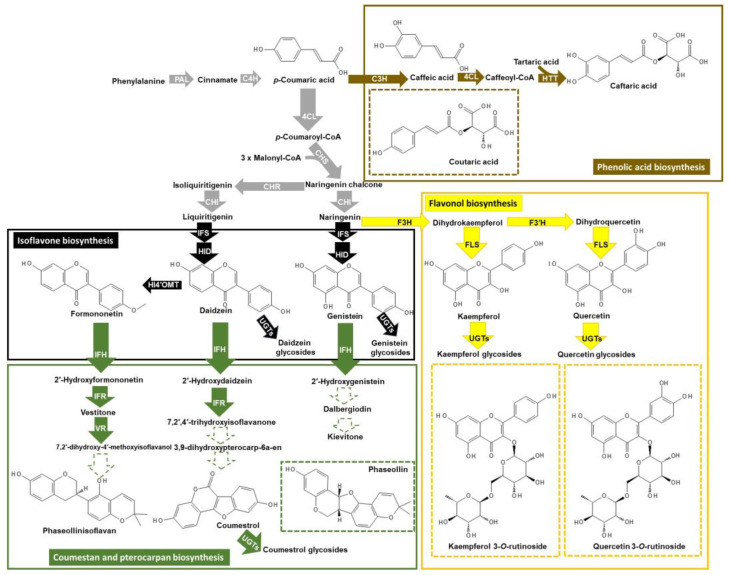
Proposed phenylpropanoid metabolism in *Phaseolus vulgaris* subjected to drought. This biochemical scheme was adapted from models of isoflavone, coumestan, pterocarpan, flavonol glycoside, and phenolic acid biosynthesis pathways from previous sources [13,17,18,19,20] and incorporates some of the corresponding metabolites that are known to occur in *P. vulgaris* [19,21,22]. Early phenylpropanoid biosynthesis steps that are shared between the phenolic acid, isoflavone, and flavonol pathways are represented by enzymatic steps within grey arrows. Black arrows denote metabolic steps associated with the biosynthesis of isoflavones (e.g., daidzein), whereas green arrows denote metabolic steps leading to the production of coumestans and pterocarpans. Arrows with a dashed green outline represent unknown reactions for the biosynthesis of phaseollinisoflavan, coumestrol, and kievitone. Enzymatic reactions for phenolic acid biosynthesis are represented by the brown arrows, whereas yellow arrows represent flavonol glycoside biosynthesis steps. Metabolites encased within green and brown dashed outline boxes respectively represent a pterocarpan and a phenolic acid for which their precise biosynthetic steps are not shown. Flavonol glycosides within yellow dashed outline boxes represent examples of kaempferol and quercetin glycosides that accumulate in *P. vulgaris* [19]. Abbreviations include: 4CL, 4-coumaroyl:coenzyme A ligase; C3H, coumarate 3-hydroxylase; C4H, cinnamate 4-hydroxylase; CHI, chalcone isomerase; CHR, chalcone reductase; CHS, chalcone synthase; F3H, flavanone 3-hydroxylase; F3′H, flavonoid 3′-hydroxylase; FLS, flavonol synthase; HI4′OMT, 2-hydroxyisoflavanone 4′-*O*-methyltransferase; HID, 2-hydroxyisoflavanone dehydratase; HTT, hydroxycinnamoyl-CoA: tartaric acid hydroxycinnamoyl transferase; IFH, isoflavone 2′-hydroxylase; IFR, isoflavone reductase; IFS, isoflavone synthase; PAL, phenylalanine ammonia-lyase; UGT, UDP-glucose dependent glycosyltransferase; VR, vestitone reductase. Images of metabolite structures were drawn with the online software PubChem Sketcher, v2.4 (https://pubchem.ncbi.nlm.nih.gov//edit3/index.html, accessed on 14 February 2024) [23].

Flavonoids are C_6_-C_3_-C_6_ derivatives of the phenylpropanoid pathway with a core chemical skeleton of aromatic A and B rings that are connected to an oxygen-containing heterocyclic C ring (Figure 1). Flavonoids consist of subclasses such as anthocyanins, flavonols, and flavones that are distinguished by the degree of saturation of the C ring, the precise C ring carbon (C2 versus C3) that is linked to the B ring, and the hydroxylation pattern of the molecule [14,15]. Hydroxycinnamic acids (e.g., *p*-coumaric acid) are phenolic acids derived from the phenylpropanoid pathway that consist of a C6 aromatic ring tethered to a C3 moiety (Figure 1). Flavonols (e.g., kaempferol and quercetin) occur across the plant kingdom and are often glycosylated [13], whereas isoflavones are legume-specific, and their presence in these plants is a balance between their aglycone (e.g., daidzein and genistein) and glycoside forms (e.g., daidzin and genistin) [15]. Phenylalanine ammonia-lyase is the initial step in the phenylpropanoid pathway and provides carbon skeletons for the various flavonoid branch pathways (Figure 1), as well as the cell wall structural polymer lignin. In grasses, tyrosine ammonia-lyase can operate as the first step in the phenylpropanoid pathway [16]. For many plant species, the increased gene expression and/or activity of phenylalanine ammonia-lyase as well as downstream phenylpropanoid pathway steps are hallmark responses to drought (Figure 1), but the accumulation of a specific class of phenylpropanoids (e.g., flavonoids versus lignin) is dependent on specific transcription factors and/or microRNAs that upregulate or downregulate these competing phenylpropanoid branch pathways [16,17]. 

Currently, there is some debate as to whether phenylpropanoid accumulation is a universal metabolic response to drought in plants. A transcriptome analysis of the roots of BAT477 (a drought-tolerant common bean line) revealed the presence of *ISOFLAVONE SYNTHASE* transcripts, pointing to the upregulation of the isoflavone biosynthesis pathway with drought [24]. Moreover, isoflavone pathway gene transcripts and isoflavones (e.g., genistein 7-*O*-glucoside) accumulate in the shoots of drought-tolerant alfalfa [25]. Further analysis is required to determine whether isoflavone metabolism is altered in common bean plants grown under limiting soil water. Apart from isoflavones, there is evidence that flavonol glycoside concentrations are augmented in plants subjected to drought. Previous research revealed a greater presence of anthocyanins, as well as kaempferol glycosides and quercetin glycosides, within five days of water being withheld from *Arabidopsis thaliana* (L.) Heynh. seedlings [26]. With respect to legumes, a 1.1-fold increase in quercetin glycoside concentrations was reported in white clover (*Trifolium repens* L.) leaves after nine weeks at 10% soil moisture content as compared to the control (i.e., 38% soil moisture content) [27]. Conversely, drought increased kaempferol glycoside concentrations in *T. repens* × *Trifolium uniflorum* L. hybrids [28]. The cessation of watering induced the accumulation of various flavones (e.g., luteolin 7-*O*-glucoside) and phenolic acids (e.g., *p*-coumaric acid) in *Achillea pachycephala* Rech. f. leaves [29]. There is also evidence demonstrating a lower abundance of phenylpropanoids in drought-stressed plants. For example, flavonoid glycoside and phenolic acid profiles were not widely affected in *Ligustrum vulgare* L. leaves after 8 weeks of growth under a watering regimen that supplied 40% of the evapotranspiration requirement relative to plants supplied with full water [30]. Similarly, flavonoid and phenolic acid concentrations were little affected in the leaves of *Amaranthus cruentus* L. when water was withheld from greenhouse-cultivated plants for 6 days [31]. Moreover, Rao et al. [32] determined that a greater proportion of leaf flavonoid constituents were elevated in a drought-tolerant sour orange and to a higher magnitude after 18 days of drought, whereas fewer metabolites were impacted in the leaves of a drought-sensitive lemon. Thus, there appears to be a discrepancy as to whether phenylpropanoids accumulate with drought. Marchin et al. postulated that withholding water for the simulation of drought is not representative of the slow decline of water that occurs in natural soils with a decline in precipitation [33]. Thus, it is imperative to determine whether altered flavonoid and related phenylpropanoid profiles occur in *P. vulgaris* under simulated drought conditions that are based on restricted watering and not cessation.

Prior research has shown that the isoflavones daidzein and genistein, and their derivatives coumestrol and phaseollinisoflavan, amass in the leaves of the white bean recombinant inbred line (RIL) BT6 when challenged with the causal agent of common bacterial blight (CBB), whereas this metabolic phenomenon is absent in its genetic relative BT44, a CBB-susceptible RIL [19]. Both RILs were derived from OAC Seaforth and OAC Rex, with the latter parent containing introgressively hybridized *Phaseolus acutifolius* (tepary bean) genes [34], including three genes that match expressed sequence tags from drought-stressed *P. acutifolius* leaves [35]. As highlighted by Moghaddam et al. [36], *P. acutifolius* originated within the arid regions of Mexico, and in addition to its resistance to CBB, it is also tolerant to heat and drought. Given the above-mentioned varying metabolic profiles in BT6 and BT44 when challenged by biotic stress and their shared *P. acutifolius* ancestry, our aim was to investigate whether phenylpropanoid profiles are altered in white bean plants when subjected to a soil water deficit, and whether these responses differed between BT6 and BT44.

## 2. Materials and Methods

### 2.1. Plant Material and Cultivation

The F6 progeny of two white bean RILs, BT6 and BT44, were produced in a greenhouse [19]. Prior to their use in this study, seeds of both RILs were stored at 4 °C in a humidity-controlled chamber. To evaluate the impact of soil water deficit on both RILs, plants were cultivated under 75% relative soil water content (RSWC), and their biomass and metabolite profiles were assessed relative to plants cultivated under 15% RSWC (i.e., severe drought). The 15% RSWC was chosen for this study as it was previously shown to elicit severe drought stress on apple rootstocks, cotton, and red clover [37,38,39]. Plants were grown in 1.45-L high-density polyethylene (HDPE) pots (Berlin Packaging^®^, Chicago, IL, USA) without drainage holes, containing two parts by volume granitic “B sand” (Hutcheson Sand & Mixes, Huntsville, ON, Canada) and 1 part peat-based potting mix (PGW, Sun Gro Horticulture Canada Ltd., Seba Beach, AB, Canada). Targeted RSWC levels were achieved using a semi-automated daily pot weighing procedure [40]. The maximum water-holding capacity of the soil at 100% RSWC was determined as the difference in mass between a pot fitted with drainage holes, watered to free drainage, and allowed to drain for 48 h, and that same pot containing soil after the soil had been oven-dried to constant weight. That total mass of water at 100% RSWC was multiplied by either 0.75 or 0.15 and added to the dry soil plus pot weight to establish the target weights for the 75% RSWC and 15% RSWC treatments, respectively.

The following procedure was used to investigate the impact of varying the RSWC on white bean growth and phenylpropanoid metabolism. Two BT6 seeds were sown in each of 48 pots. A second set of 48 pots were sown with BT44 seeds. Before sowing, each pot received 100 mL of fertilizer solution containing 10 g L^−1^ 20-20-20 (N-P-K) water-soluble fertilizer (Master Plant Prod Inc., Brampton, ON, Canada), 2.5 g L^−1^ calcium nitrate (Master Plant-Prod Inc.), and 2.5 g L^−1^ magnesium sulfate (Plant Products, Leamington, ON, Canada). At the time of sowing, the soil moisture content was 83% RSWC. For each RIL, 12 pots were transferred to each of four separate experimental benches located in a large, controlled growth room within the Crop Sciences Building at the University of Guelph and arranged in a randomized complete block design. Within each bench, plants were rotated once per day to limit positional effects within that experimental block of the growth room. Plants within each of the four experimental blocks were cultivated at 25 °C under a 16-h photoperiod and at 20 °C for an 8-h dark period under a constant relative humidity of 55%. The overhead lighting system consisted of T8 LED and incandescent bulbs delivering an irradiance of 265 µmol m^−2^ s^−1^ photosynthetically active photon flux. All pots were irrigated with 100 mL on each of the first four days after sowing. On the fourth day post-emergence, each pot was thinned to a single plant and fitted with a HDPE lid (Berlin Packaging^®^) to limit the evaporation of water from the soil surface. Each lid had two 1-cm diameter holes—one to accommodate the plant stem, and another to permit water additions. On the seventh day post-emergence, fertilizer was again added to each pot in the same amount as described above. Potted plants were weighed daily and irrigated with a semi-automated gravimetric system [40]. All pots were maintained at 75% RSWC until 14 days post-emergence. Bamboo poles were inserted into each pot to support the vines of the plants, and a sachet of predatory mites (BIOBEST GROUP NV, Leamington, ON, Canada) was placed near each plant as a biological control against insects. The masses of the poles and sachets were added to the target weights of the pots programmed into the semi-automated watering system.

On day 14 post-emergence (i.e., day 0 of the varying RSWC experiment), six plants of each RIL within each of the four experimental replicate benches were switched to 15% RSWC (i.e., severe drought) using the above-mentioned pot weighing strategy, with the remaining pots continually cultivated under 75% RSWC (i.e., control). Immediately thereafter, one plant of each RIL and RSWC treatment combination was sampled from each of the four experimental replicates, and vegetative tissues were harvested as outlined below. Each sampled plant was dissected into leaf, stem, and root fractions. Unifoliate and trifoliate leaves were sampled in an alternating pattern to amass approximately the same number of leaves in each of two separate pools. The fresh weight (FW) of each pool of leaves was taken; one of the leaf pools was flash frozen in liquid N_2_ and transferred to a −80 °C freezer for downstream metabolite analysis. The plant’s roots were washed to remove soil residue, and then blot-dried with paper towels. Thereafter, the root tissue was dissected into two approximate halves by cutting along the longitudinal axis of the primary root. The FW for each of the two root sections was taken, and one of these was flash frozen in liquid N_2_ and then transferred to a −80 °C freezer for downstream metabolite analysis. The stem, devoid of leaves and petioles, was analyzed for FW. For dry weight analysis (DW), the remaining root, stem, and leaf materials that were not frozen were transferred to separate paper bags and placed in a drying oven at 60 °C until fully dried. The aforementioned sampling procedure for leaves and roots from each plant permitted the rapid freezing of one of the two fractions corresponding to each organ as a strategy to preserve the metabolite integrity of harvested vegetative tissues [41]. Given that approximately half of each of the leaves and roots of each plant were frozen on the day of sampling and not dried, we estimated the DW of each root and leaf sample by extrapolation to the amount of original organ material collected. For this, the DW of the whole sample (i.e., dried and frozen sub-samples) was calculated as the DW of the dried sub-sample divided by the ratio of the original fresh weight of the sub-sample dried after harvest: whole sample FW. A similar sampling strategy to that described above was used on days 3, 6, 9, and 12 of the varying RSWC cultivation experiment. 

### 2.2. Metabolite Analysis

Phenolic acids, flavonoids, and their related compounds were extracted from 0.1 g of frozen white bean leaf or root pulverized material. The frozen powder was combined with 0.5 mL of acidified methanol (45% methanol [*v*/*v*], 5% [*v*/*v*] acetic acid in Milli-Q water) containing 11.5 µM naringin (internal standard) and then resuspended by vortex followed by gentle inversion on a nutator for 20 min. The extract was clarified by centrifugation at 10,000× *g* for 10 min, and the supernatant was collected. The pellet was re-extracted twice with acidified methanol, as described. Thereafter, the supernatants of all three extractions were pooled and filtered through a 0.45 µm PTFE filter (Mandel Scientific Company Inc., Guelph, ON, Canada) and analyzed by UPLC-MS/MS as described previously [42]. For the UPLC-MS/MS analysis, a 2 µL aliquot of the filtered extract was injected onto a Kinetex XB-C18 100Å column (100 × 4.6 mm, 2.6 µm, Phenomenex, Torrance, CA, USA) connected to a Vanquish ™ Flex Binary UPLC System (Thermo Fisher Scientific, Waltham, MA, USA). The column was pre-equilibrated with solvent A (0.1% formic acid [*v*/*v*] in Milli-Q water) at a flow rate of 0.7 mL min^−1^. The column temperature was set to 40 °C. Phenylpropanoids were eluted with a gradient of solvent B (94.4% methanol, 5% acetonitrile, 0.1% formic acid; [*v*/*v*/*v*]) at the same flow rate. The elution gradient was as follows: 0–12% solvent B (0–5 min), 12–23% solvent B (5–15 min), 23–50% solvent B (15–30 min), 50–80% solvent B (30–40 min), and 80–100% solvent B (40–42 min). The gradient was held at 100% B for 3 min before returning to 0% solvent B over a 1 min linear gradient, and then held at 0% solvent B for an additional 6 min prior to the subsequent injection. An in-line Thermo Fisher Scientific Q-Exactive Orbitrap MS (Waltham, MA, USA) in negative electrospray ionization mode was used to detect metabolite masses and fragmentation patterns, as outlined by Cox et al. [19]. Thermo Scientific Q-Exactive Orbitrap MS in negative electrospray ionization mode was used to detect metabolite parent and fragment ions (normalized collision energy = 30), as outlined by Cox et al. [19]; these data were analyzed with Thermo FreeStyle™ 1.7 software (Thermo Fisher Scientific). The annotation of the eluted metabolites (Supplementary Information, Table S1) was based on a comparison of their MS/MS fragmentation data to those of isoflavones (and their derived metabolites), phenolic acids, and flavonol glycosides that are present within an in-house library or fragmentation ion patterns described previously [19,43]. 

### 2.3. Statistical Analysis

For all biomass, water use and metabolite profile data, separate analyses of variance (ANOVA) were performed in R [44] to determine significant differences at the α = 0.05 level. For each ANOVA, data were from 80 different *P. vulgaris* samples, representing both RILs, both RSWC treatments, and five samplings over the 12-day time course across four controlled environment room blocks. Prior to any means comparisons, the data was assessed for independence of errors, and a Shapiro’s Wilk test was used to test for normality. For data that was not normally distributed, a lognormal distribution was applied prior to any statistical analysis. The homogeneity of the data was verified by a Levene’s test on the data residuals. A Tukey-Kramer adjustment was applied to a multiple means comparison using the EMMeans function in R to test the significant differences within the data for all 80 *P. vulgaris* samples representing both RILs cultivated at 15% and 75% RSWC and sampled at 0, 3, 6, 9, and 12 days of the cultivation period for four separate experimental replicates. This was done for each of the leaf, stem, root, or whole plant data that were assessed for one or more of the following: changes in cumulative water use, biomass (FW and DW), and individual metabolite profiles.

## 3. Results

### 3.1. Effect of a Reduction in Irrigation Water Supply on Plant Growth Dynamics and Water Use 

**Figure 2:**
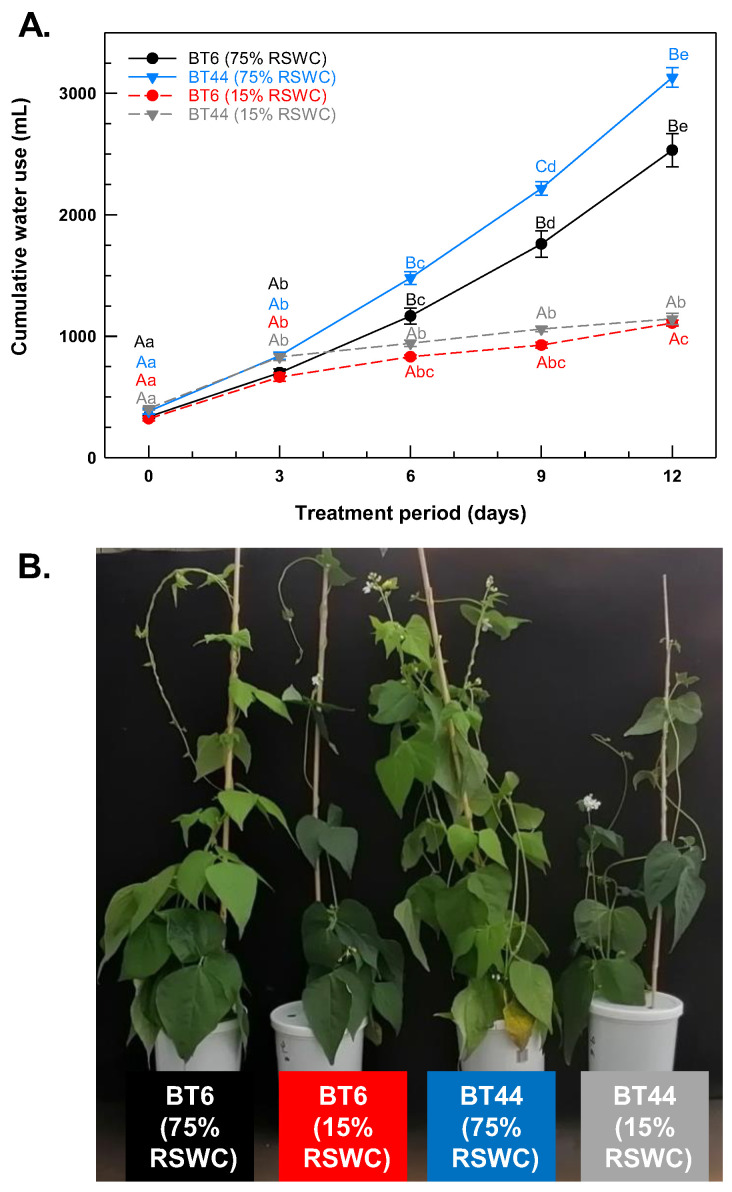
(**A**) Cumulative water use of white bean RILs BT6 and BT44 cultivated under 75% relative soil water content (RSWC) and 15% RSWC. Each datum represents the cumulative water use mean ± standard error of four experimental replicates. Means sharing the same letters are not statistically different. Uppercase letters indicate significant differences across the treatments within a single sampling day. Lowercase letters indicate significant differences within a treatment across the 12-day experimental period. Black and blue lettering correspond to the proximal BT6 and BT44 75% RSWC treatment data, respectively. Red and grey lettering correspond to the proximal BT6 and BT44 15% RSWC treatment data, respectively. (**B**) Representative images of BT6 and BT44 plants sampled from 75% RSWC and 15% RSWC on day 12 of the cultivation period.

Plants were cultivated under 75% RSWC until day 14 post-emergence and then subjected to severe drought (15% RSWC) or left at 75% RSWC (control; well-irrigated) for the following 12 days. To confirm that less water was acquired under severe drought relative to the control irrigation treatment, cumulative water use was assessed over the 12-day time course (Figure 2A). There were no differences among the treatments with respect to the amount of water used over the initial three days, but thereafter, water use was reduced in the 15% RSWC treatment. On day 12, the cumulative water use was respectively 56% and 64% lower in BT6 and BT44 plants held under severe drought relative to well-watered plants. Overall, plants held at 15% RSWC for 12 days tended to have a thinner canopy than plants sampled from the 75% RSWC treatment (Figure 2B).

**Figure 3:**
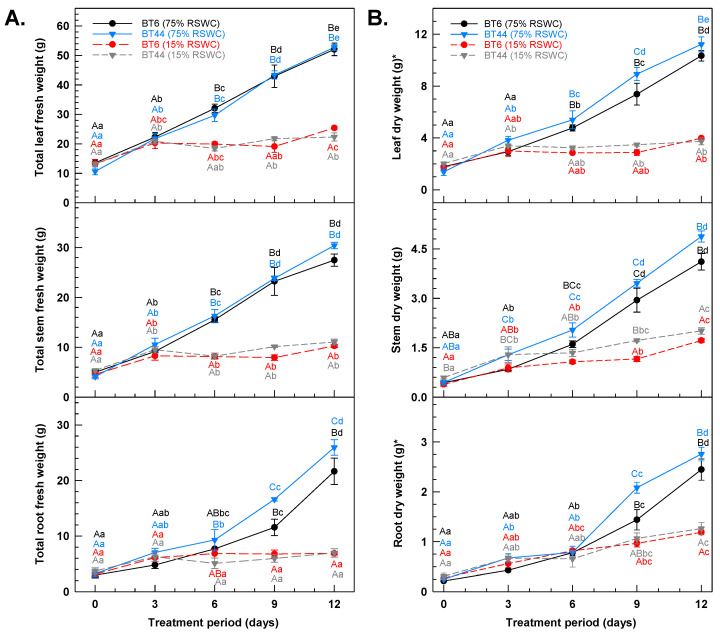
Changes in the (**A**) total fresh weight (FW) and (**B**) total dry weight (DW) of leaves, stems, and roots of white bean plants cultivated under 75% relative soil water content (RSWC) and 15% RSWC. The top, middle, and bottom plots within each panel represent data corresponding to leaf, stem, and root biomass, respectively. The asterisk in the y-axis label of the total leaf DW plot denotes an extrapolation of the data for the leaf DW per plant; this was calculated by dividing the DW by the proportion of the total leaf FW that was dried after harvest. A similar calculation was performed for the root DW per plant; an asterisk notation in the y-axis label is used to note that the represented scale is for the extrapolated data. The stem DW was based on the drying of the total stem material following excision of leaves and petioles. Each datum within a plot represents the mean ± standard error of the total biomass of the corresponding organ across four experimental replicates. Means sharing the same letters are not statistically different. Uppercase letters indicate significant differences across the treatments within the sampling day. Lowercase letters indicate significant differences within a treatment across the 12-day experimental period. Within each plot, black and blue lettering correspond to the proximal BT6 and BT44 75% RSWC treatment data, respectively; red and grey lettering correspond to the proximal BT6 and BT44 15% RSWC treatment data, respectively.

Changes in the FW of vegetative tissues as a function of cultivation period were also determined (Figure 3A). On average, the leaf FW of both RILs increased by 340% in well-watered plants over the 12-day experiment. By contrast, the FW of BT6 leaves was elevated by 54% by the third day of drought and remained unchanged thereafter, whereas a maximal increase of 40% was evident in BT44 leaves, although, this was delayed until day 6. For both RILs, an 80% average increase in stem FW occurred within three days of exposure to drought but was unchanged thereafter. Much larger increases in stem FW were apparent in the well-watered plants, with 167% and 174% more stem weight amassed per plant in BT6 and BT44, respectively, relative to the 15% RSWC treatment. Similar trends were apparent with respect to the impact of drought on the increase in root FW per plant. It is important to note that these root FW alterations were first evident by day 6 of the 75% RSWC treatment, whereas root FW remained unchanged with severe drought.

Changes in leaf, stem, and root DWs of both RILs under each RSWC treatment were also impacted by the RSWC (Figure 3B). All well-watered plants accumulated DW over the 12-day time course. Specifically, 480-1040% increases were apparent for the DW of all vegetative tissues of BT6 plants, whereas approximately 700–1000% more DW was evident in BT44 plants over the 12-day period. By comparison, much less leaf DW accumulated with severe drought, with increases of 84% and 130% in BT44 and BT6, respectively. Similarly, the final stem DW for all drought-stressed plants was on average 59% less than that of the control plants. In these same plants, minor changes in the root DW were not evident until the sixth and ninth days of drought in BT6 and BT44, respectively.

It is worth noting that transition to reproductive growth was evident on the ninth day of the experiment, regardless of the RIL and RSWC treatment (Supplementary Information, Figure S1). The total FW of flowers (including undeveloped floral buds) was at least 116% greater on day 9 of the 75% RSWC treatment than that measured for flowers formed under severe drought. Thereafter, the floral FW increased by 85–175%, with the largest changes evident at 75% RSWC.

### 3.2. Impact of Relative Soil Water Content on the Phenylpropanoid Profiles of White Bean Roots

**Figure 4:**
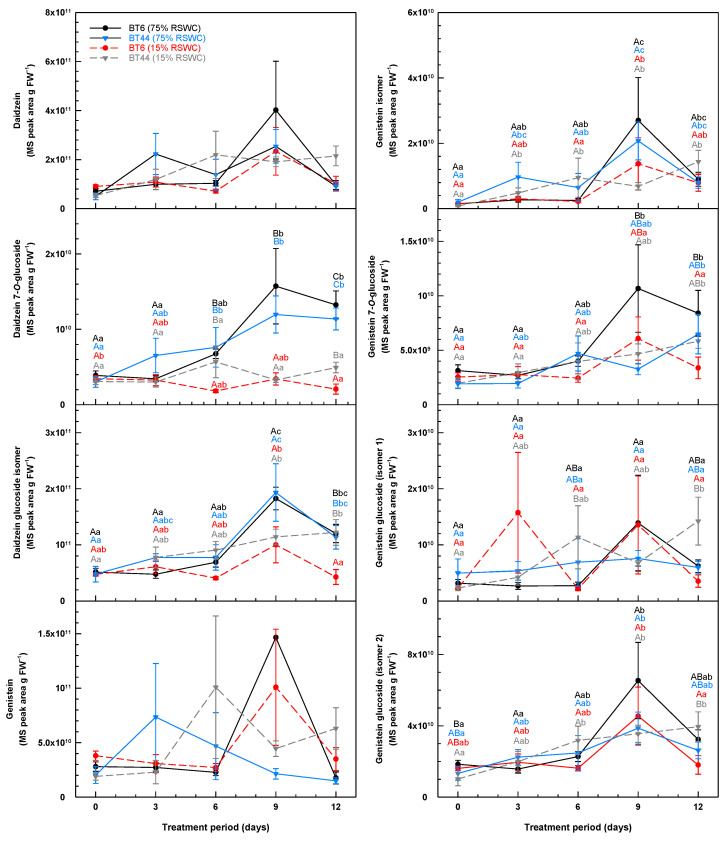
Temporal alterations in the root isoflavone and isoflavone glycoside profiles during cultivation of white bean RILs BT6 and BT44 under 75% relative soil water content (RSWC) or 15% RSWC. For each metabolite, the concentration is based on the MS peak area associated with the intensity of the metabolite’s parent ion (*m*/*z*) divided by the precise mg of root tissue extracted and then extrapolated and expressed as MS peak area g FW^−1^. Each datum within a metabolite plot represents the mean ± standard error of four experimental replicates. Means sharing the same letter are not statistically different. Uppercase letters indicate significant differences across the RILs and their treatments within a single sampling day. Lowercase letters indicate significant differences within a treatment across the 12-day treatment period. Black and blue lettering correspond to the proximal BT6 and BT44 75% RSWC treatment data, respectively; red and grey lettering correspond to the proximal BT6 and BT44 15% RSWC treatment data, respectively. For the daidzein and genistein panels, the data are not accompanied by statistical letters, as in either case there was no significant difference across any of the compared means.

**Figure 5:**
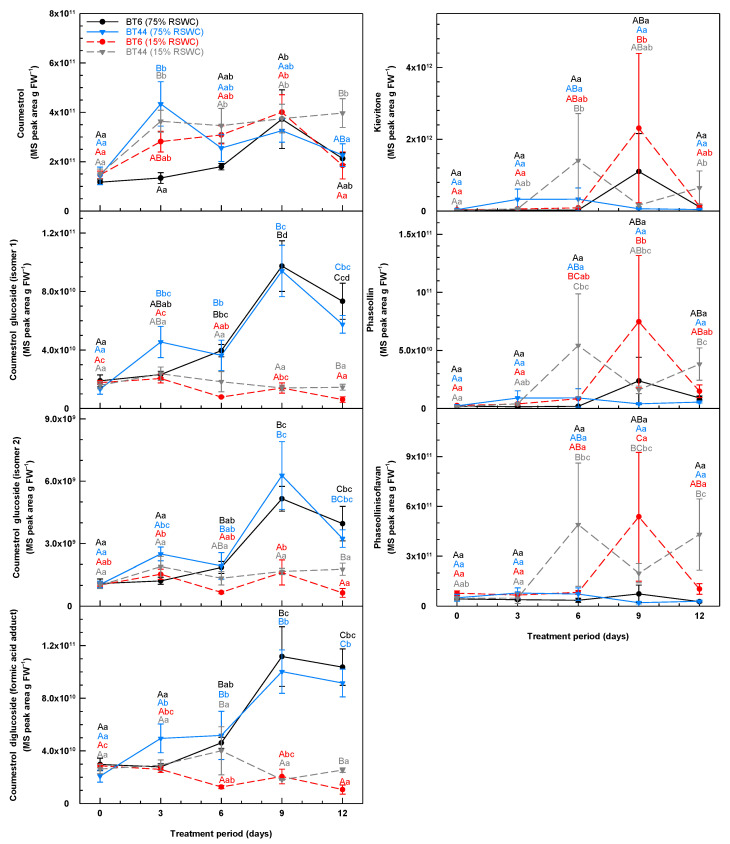
Temporal alterations in the root concentrations of coumestans and other isoflavone derivatives during cultivation of white bean RILs BT6 and BT44 under 75% relative soil water content (RSWC) or 15% RSWC. For each metabolite, the concentration is based on the MS ion peak area associated with the intensity of the metabolite’s parent ion (*m*/*z*) divided by the precise mg of root tissue extracted and then extrapolated and expressed as MS peak area g FW^−1^. Each datum within a metabolite plot represents the mean ± standard error of four experimental replicates. Means sharing the same letter are not statistically different. Uppercase letters indicate significant differences across the RILs and their treatments within a single sampling day. Lowercase letters indicate significant differences within a treatment across the 12-day treatment period. Black and blue lettering correspond to the proximal BT6 and BT44 75% RSWC treatment data, respectively; red and grey lettering correspond to the proximal BT6 and BT44 15% RSWC treatment data, respectively.

The roots of both RILs contained many isoflavones (including their glycosides) (Figure 4), coumestans, and pterocarpans (Figure 5). Although there were fluctuations with the cultivation period, there was no clear effect of RIL or severe drought on the relative levels (expressed as MS peak area per g FW) of daidzein, genistein and its related isomer, and genistein glucoside (isomer 2) (Figure 4). The concentrations of other isoflavone glycosides were altered by the RSWC in one or both RILs on day 9 of the time course and/or thereafter. On day 12, the roots of drought-stressed BT6 had 64% on average less of a daidzein glucoside isomer than the BT6 control and all BT44 plants. Moreover, the genistein 7-*O*-glucoside concentration in BT6 was 149% greater at this same time when cultivated at 75% RSWC relative to the lower water supply regimen. Finally, daidzein 7-*O*-glucoside levels in both RILs were on average 3.1-fold greater by day 9 of cultivation at 75% RSWC relative to 15% RSWC. 

Temporal fluctuations in the root concentrations of coumestrol occurred with cultivation, with some RIL and/or RSWC-specific alterations (Figure 5). By day 3, there was an average 175% elevation of root coumestrol in all BT44 plants, whereas this change was delayed for an additional six days in drought-stressed BT6 plants. Thereafter, for most plants, the coumestrol concentrations returned to the levels apparent at the beginning of the experiment, whereas the accumulated coumestrol levels were sustained in BT44 with severe drought, albeit the final concentration on day 12 was similar to that detected in the roots of the control plants. There were maximal increases in the root concentrations of coumestrol glucoside isomers and the putative formic acid adduct of coumestrol diglucoside by day 9 of the control treatment, whereas no changes were evident in response to drought. Overall, the concentrations of these coumestrol glycosides were 220–590% greater in the well-watered plants of both RILs than in the drought-stressed plants. Apart from coumestans, other isoflavone derivatives were detected in the roots of the white bean RILs, specifically kievitone, phaseollin, and phaseollinisoflavan. There was no clear effect of drought on root kievitone levels. The root concentrations of phaseollin increased as much as 24.6-fold and 27.5-fold with drought in BT44 and BT6, respectively. BT44 had approximately 5- and 6-fold more of this metabolite on days 6 and 12 of drought, respectively, than the concentrations within the roots of well-watered plants. In BT6, there was 3.5-fold more phaseollin on day 6 of drought relative to control plants. Finally, fluctuations in the concentrations of phaseollinisoflavan occurred in BT44 under severe drought but not in BT6. Nonetheless, by day 9 of drought, there was 630% and 860% more phaseollinisoflavan in BT6 and BT44 plants, respectively, as compared to well-watered plants; these levels were sustained in BT44 but not BT6 roots.

### 3.3. Impact of Relative Soil Water Content on the Phenylpropanoid Profiles of White Bean Leaves

**Figure 6:**
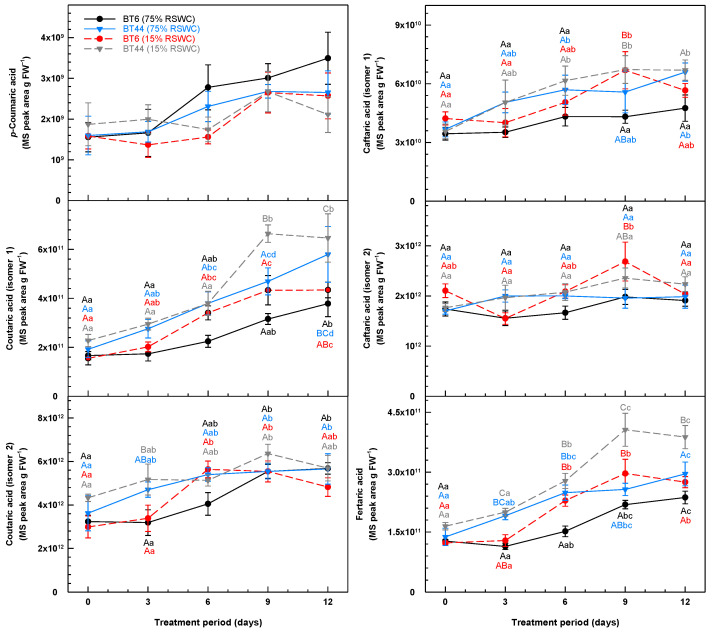
Temporal alterations in the leaf concentrations of phenolic acids during cultivation of white bean RILs BT6 and BT44 under 75% relative soil water content (RSWC) or 15% RSWC. Each phenolic acid concentration is based on the MS peak area associated with the intensity of the metabolite’s parent ion (*m*/*z*) divided by the precise mg of leaf tissue extracted and then extrapolated and expressed as MS peak area g FW^−1^. Each datum within a metabolite plot represents the mean ± standard error of four experimental replicates. Means sharing the same letter are not statistically different. Uppercase letters indicate significant differences across the RILs and their treatments within a single sampling day. Lowercase letters indicate significant differences within a treatment across the 12-day treatment period. Black and blue lettering correspond to the proximal BT6 and BT44 75% RSWC treatment data, respectively; red and grey lettering correspond to the proximal BT6 and BT44 15% RSWC treatment data, respectively. For the *p*-coumaric acid panel, the data are not accompanied by statistical letters as there was no significant difference across any of the compared means.

In addition to isoflavones and coumestans, white bean leaves contained a wide array of flavonol glycosides and phenolic acids. The concentrations of *p*-coumaric acid and other phenolic acids were increased in the leaves of the white bean RILs during the cultivation period (Figure 6). Six phenolic acids were detected, including *p*-coumaric acid, two coutaric acid isomers, two caftaric acid isomers, and fertaric acid. Coutaric acid, caftaric acid, and fertaric acid are derived from the conjugation of tartaric acid to *p*-coumaric acid, caffeic acid, and ferulic acid, respectively. There was no consistent effect of drought on the concentrations of *p*-coumaric acid and coutaric acid (isomer 2). Drought affected the profiles of the other phenolic acids, although this was transient in some cases. Drought yielded 42% more coutaric acid (isomer 1) in BT44 leaves on day 9 relative to the control treatment. At this same time, there was 55% more caftaric acid (isomer 1) in the leaves of drought-stressed BT6 as compared to the leaves of their well-watered counterparts. Moreover, on day 9 alone, there was 35% more caftaric acid (isomer 2) in BT6 at 15% RSWC relative to the control. Lastly, drought induced the accumulation of fertaric acid in both RILs, albeit for BT6, the increased concentration relative to the control was apparent on days 6 and 9. Conversely, on day 9 fertaric acid was as much as 58% greater in BT44 leaves under drought than at 75% RSWC, and this effect was sustained until the end of the cultivation period. 

Isoflavones and some of their derivatives were detected within white bean leaves, although it is unclear whether their concentrations were affected by drought, as statistical comparisons of the means were not possible given the complex distribution of the data (Supplemental Information, Figure S2). Except for kievitone and coumestrol, there was little evidence for the presence of the other isoflavone derivatives within the leaves during the initial six days of the cultivation period. Genistein and its isomers were more abundant toward the end of the cultivation period. Flavonol glycosides were detected in white bean leaves, with some solely present in one RIL. Twelve quercetin glycosides were detected in white bean leaves, including five compounds that were not found or negligible in BT44 plants (Supplemental Information, Figure S3). These were quercetin 3-*O*-galactoside, quercetin xyloside, quercetin dixyloside (isomer 2), quercetin glucoside-xyloside (isomer 1), and quercetin rutinoside-xyloside (isomer 2). In BT6 leaves, most of these quercetin compounds were present at 53–900% greater concentrations by day 12 at 75% RSWC relative to 15% RSWC. About 80% more quercetin 3-*O*-glucuronide was detected in BT44 leaves of the control treatment as opposed to the drought treatment. All other quercetin glycosides were little affected by RSWC.

**Figure 7:**
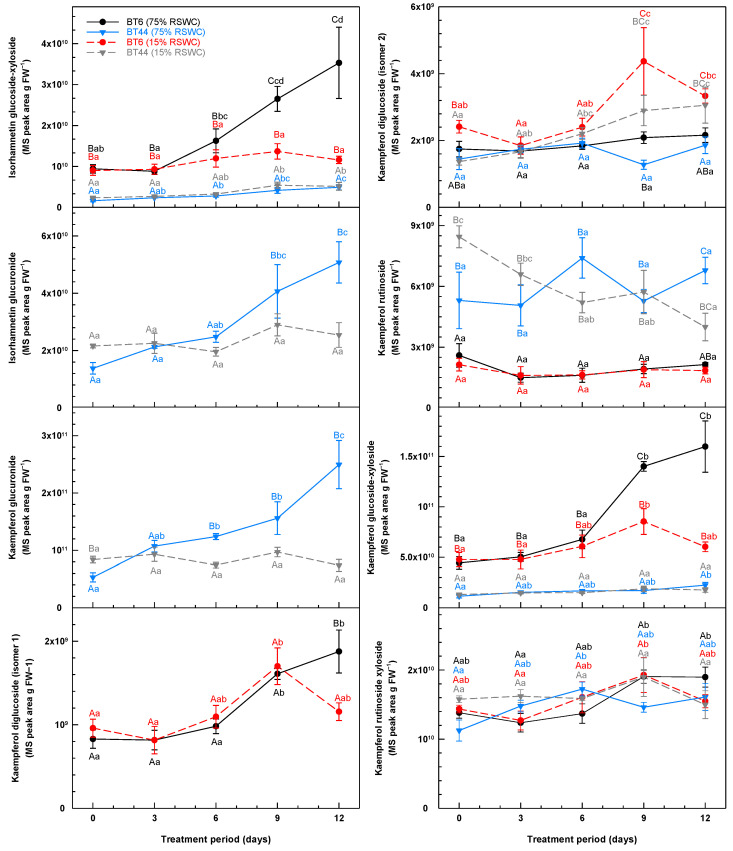
Temporal alterations in the concentration of leaf isorhamnetin glycosides and kaempferol glycosides during cultivation of white bean RILs BT6 and BT44 under 75% relative soil water content (RSWC) or 15% RSWC. Each metabolite concentration is based on the MS ion peak area associated with the intensity of the metabolite’s parent ion (*m*/*z*) divided by the precise mg of leaves extracted and then extrapolated and expressed as MS peak area g FW^−1^. Each datum within a metabolite plot represents the mean ± standard error of four experimental replicates. Means sharing the same letter are not statistically different. Uppercase letters indicate significant differences across the RILs and their treatments within a single sampling day. Lowercase letters indicate significant differences within a treatment across the 12-day treatment period. Black and blue lettering correspond to the proximal BT6 and BT44 75% RSWC treatment data, respectively; red and grey lettering correspond to the proximal BT6 and BT44 15% RSWC treatment data, respectively.

The leaf profiles of various isorhamnetin glycosides and kaempferol glycosides were impacted by RSWC and/or RIL (Figure 7). Overall, isorhamnetin glucoside-xyloside concentrations were unchanged in all BT44 plants. After day 6, there was as much as 2-fold more of this flavonol glycoside in the leaves of control BT6 plants than drought-stressed plants. Isorhamnetin glucuronide was absent in BT6, and its concentration was stable in BT44 leaves with drought, whereas in the control treatment a doubling was evident in this same RIL by day 12. Of the six kaempferol glycosides that were detected, kaempferol glucuronide was solely present in BT44, whereas kaempferol diglucoside (isomer 1) was detected in BT6 but not BT44. The majority of these kaempferol metabolites seemed to accumulate in well-watered plants. Kaempferol glucuronide levels were elevated in BT44 leaves at 75% RSWC and unchanged with drought. In BT6, kaempferol diglucoside (isomer 1) doubled in concentration by day 9 of cultivation, regardless of treatment. After an additional three days, there was 62% more of this metabolite in well-watered plants relative to drought-stressed plants. Kaempferol rutinoside concentrations were much lower in BT6 than in BT44 and were unaffected by the RSWC. By contrast, a near halving of this metabolite’s levels occurred in BT44 with severe drought as opposed to no change for plants held at 75% RSWC. In addition, a near 2-fold accumulation of kaempferol glucoside-xyloside occurred in BT6 leaves at 75% RSWC, whereas a minor accumulation was evident with drought. Unlike all the other kaempferol glycosides, severe drought amplified leaf kaempferol diglucoside (isomer 2) concentrations. Regardless of RIL, by day 9 of cultivation, the concentrations of kaempferol diglucoside (isomer 2) in drought-stressed plants were approximately double those of the plants under 75% RSWC. These differences were sustained thereafter.

## 4. Discussion

Here, we tested the impact of severe drought on the alterations in biomass and phenylpropanoid metabolism of white bean RILs derived from a cross of OAC Rex and OAC Seaforth, the former containing genes from the drought-resistant tepary bean [34,35]. To simulate severe drought, we used a pot weighing strategy to achieve a 15% RSWC. This promotes a slow loss of soil moisture by evapotranspiration, which more closely resembles what occurs in nature. This is distinct from sub-optimal approaches where drought is imposed by the abrupt termination of water irrigation or the application of polyethylene glycol to soils, the latter of which can induce a rapid adjustment in the plant’s osmotic potential [33,45]. The 32–40% reduction in the white bean plant DW with a severe drought is slightly greater than the 25% loss in the DW of drought-tolerant chickpea, but much smaller than the 69–77% DW loss in drought-sensitive chickpea plants [46]. In general terms, the reduction in the FW of leaves and roots of both white bean RILs at 15% RSWC matches the more than 50% reduction of these two biomass parameters in lima bean when cultivated under severe drought relative to those grown in well-watered soils [47]. Severe drought also reduced the floral FW of both white bean RILs, which could have negatively impacted seed yield in these plants relative to well-irrigated plants. It is well known that seed yield is reduced in drought-stressed legumes [5,7]. The phytochemical profiles of the white bean flowers were not analyzed, but it is likely that their phenylpropanoid signatures were also affected given that the flavonol glycoside and isoflavone profiles of other dry bean reproductive organs (i.e., seeds) are altered with a decline in water supply [48]. Although not assessed here, it can be assumed that the reduction in white bean plant biomass is due to stomatal closure leading to reduced leaf internal CO_2_ concentrations, and therefore reduced photosynthesis. Moreover, it is possible that any stomatal closure under drought coincides with ABA and ROS accumulation in the leaf guard cells of white bean plants, both of which have been shown to occur with drought in other plants [10,11]. Stomatal closure also limits the transportational loss of water from leaves. Dixon et al. found that wheat undergoes 31% to 38% less transpiration when plants are in soils containing 20% RSWC as compared to those holding 75% RSWC [49]. Reduced transpiration under a soil water deficit coincides with a drop in stomatal conductance, as has been demonstrated for several soybean genotypes cultivated at a soil moisture field capacity of 30% relative to 80% field capacity [50].

Drought had little effect on the isoflavone and isoflavone glycoside profiles within the roots and leaves of the white bean RILs investigated in this study. To a similar extent, our findings agree with previous research demonstrating little impact of drought on isoflavone profiles across the elongation zone and mature region of soybean seedling roots, whereas the root apex of drought-stressed soybean plants contained double the amount of total isoflavones under limiting water relative to their well-watered counterparts [51]. It remains to be determined whether the root apex of white bean roots accumulates isoflavones with drought. Alternatively, the lack of isoflavone and isoflavone glycoside accumulation in white bean roots could be due to their secretion to the rhizosphere. In fact, daidzein and genistein are secreted from soybean roots in response to nitrogen deficiency [52]. It is tempting to speculate that a high degree of isoflavone secretion to the rhizosphere occurs in BT6 and BT44, as their roots did not accumulate daidzein, genistein, nor genistein glucoside isomers during cultivation in water-limited soils. This contrasts with previous research that discovered substantial increases in the concentrations of isoflavones and coumestrol within the roots of 30-day-old soybean plants following their harvest and a subsequent 5-h dehydration treatment [53]. However, the rise in isoflavone content in the dehydrated soybean was likely a consequence of the more than 50% loss of root water content. Again, the magnitude and rapidity of this change is not representative of soil-bound plants exposed to a water deficit [33], but more likely due to postharvest desiccation. To this end, it is known that sugar beet roots lose water and undergo respiratory metabolism leading to carbohydrate depletion when stored under low-humidity environments [54]. The absence of an effect of soil water deficit on much of the root isoflavone profile in the white bean RILs is somewhat similar to the fact that whole plant isoflavone concentrations are greater in non-stressed *Sophora alopecuroides* L. than during moderate and severe drought [55]. A third alternative as to why isoflavone accumulation was absent in white bean roots with drought is their conversion to coumestans and pterocarpans.

In the roots of BT44 alone, there was a sustained accumulation of coumestrol with severe drought. The small change in root coumestrol concentrations with drought in white bean RILs contrasts with the nearly 160-fold increase that was detected in the leaves of BT6 during its resistance response to CBB [19]. Although the precise final two biochemical steps governing the biosynthesis of coumestrol in plants are unknown (Figure 1), the possibility remains that its putative steps (e.g., oxidoreductase and carboxyesterase; [56]) are differentially regulated with drought as opposed to the CBB-resistance mechanism in common bean. Apart from coumestrol, we observed more phaseollin and phaseollinisoflavan in the roots of both RILs with drought as compared to well-watered plants. To our knowledge, this is the first report describing the accumulation of these two pterocarpans in drought-stressed plants. Phaseollin and phaseollinisoflavan are proposed to function as phytoalexins and are known to accumulate in bean plants infected with microbes, including within the leaves of BT6 during its resistance response to CBB [19,43,57]. Like coumestrol, the production of these two phytoalexins was not altered in the leaves of BT6 or BT44 with severe drought. The possibility remains that the regulatory steps governing phytoalexin biosynthesis in the leaves of white bean plants subjected to severe drought are likely distinct from those that are involved in disease resistance.

Leaf *p*-coumaric acid profiles in both white bean RILs were little affected by drought. These findings contrast with previous research demonstrating the accumulation of *p*-coumaric acid in *A. pachycephala* leaves following the cessation of watering [29]. The lack of alterations in *p*-coumaric acid profiles in drought-stressed white bean leaves is not surprising given that this metabolite is a metabolic precursor of flavonoids, other phenolic acids, and the cell wall polymer lignin [16,58]. Although not investigated here, the lack of *p*-coumaric acid accumulation in white bean leaves during drought may be due to lignin accumulation, and hence upregulation of the phenylpropanoid pathway including phenylalanine ammonia-lyase. In fact, it is known that lignin levels are increased in the vasculature of numerous plant species with drought, as a strategy to prevent the collapse of xylem (the main water conduit in plants) [16]. Apart from *p*-coumaric acid, it is known that the total hydroxycinnamic acid concentration is increased in white clover with drought [28]. Increased concentrations of various phenolic acids such as *p*-coumaric acid, caffeic acid, and ferulic acid, as well as quercetin glycosides occur in amaranth plants exposed to 30% field capacity moisture for 30 days [59]. Similarly, our study found that white bean leaves had phenolic acid accumulation patterns in response to severe drought that were RIL-specific, although both RILs had more of the putative fertaric acid at 15% RSWC than at 75% RSWC. All three phenolic acids occur in grape berries [60], although coutaric and caftaric acid are in common bean seeds [21,22,61], and a metabolite with MS/MS signatures matching coutaric acid is present in faba bean seed pods [62]. Phenolic acids in the leaves of white beans subjected to severe drought are likely involved in the management of ROS; Król et al. found that grapevine seedlings exposed to 30% soil moisture for two weeks lost as much as 35% of their leaf phenolic acids, which coincided with a 20% decline in antioxidant capacity [63]. 

For the most part, flavonol glycoside concentrations were increased with the cultivation period in the leaves of the well-watered white bean plants investigated here. The accumulation of isorhamnetin-, quercetin-, and/or kaempferol glycosides under non-stress growth conditions fits with the buildup of flavonol glycosides during leaf elongation in *A. thaliana* [42,64] and the legume *Lotus japonicus* (Regel) K.Larsen [65]. Apart from the accumulation of kaempferol diglucoside (isomer 2) with drought, the profiles of most of the leaf flavonol glycosides, including those specific to BT6 or BT44, were unaffected. This contrasts with prior research demonstrating the accumulation of quercetin or kaempferol glycosides in drought-stressed white clover [27], although this study did not establish how the individual glycoside compounds were impacted by drought. As much as a 50% increase in various flavonol glycosides occurred when three-week-old *A. thaliana* plants were not watered for 5 days [26]. Again, this metabolic response is likely the result of rapid drying rather than a true representation of a natural soil water deficit. To this end, leaves of grapevine contain 150-590% more kaempferol 3-*O*-glucoside, quercetin 3-*O*-glucoside, and quercetin 3-*O*-glucuronide after 8 days of desiccation on sandy soils, although no comparisons were made to plants that were continuously well-watered [66]. Overall, the lack of flavonol glycoside accumulation in the white bean leaves mirrors the findings of other studies [30,67]. Moreover, four separate drought-sensitive cherry tomato cultivars did not amass any more leaf flavonol glycosides in their leaves when cultivated at 50% field capacity moisture relative to well-watered plants [67]. Conversely, these authors found 75–500% increased levels of various flavonol glycosides in the drought-tolerant cultivar ‘Zarina’. Thus, with respect to the flavonol composition, it seems that there is some degree of drought sensitivity in both white bean RILs. It is worth noting that the absence of phenolic acids and flavonol glycosides in the roots of both RILs relative to their occurrence in the leaves implies that their respective biosynthetic pathways are missing in the roots. It is possible that phenolic acid pathway genes (e.g., coumarate 3-hydroxylase) and flavonol biosynthesis genes (e.g., flavonol synthase) are missing within the roots of either white bean RIL or that their gene expression is differentially regulated by drought as compared to the leaves.

The lower flavonol glycoside profiles in the leaves of white bean RILs subjected to drought may be the consequence of the sampling strategy where young and mature leaves were pooled from each plant prior to the UPLC-MS/MS analysis. This explanation is based on the finding that flavonol glycosides tend to occur in smaller pools in young leaves nearest to the stem tip of grapevines relative to older leaves [68]. Alternatively, the lack of accumulation of flavonol glycosides in white bean leaves during severe drought could be necessary to limit flavonol-mediated stomatal opening. Previous research revealed that ROS-mediated stomatal closure is inhibited within the leaves of flavonol hyper-accumulating *A. thaliana* plants [11]. Minimal or no transpiration occurs in the leaves of drought-stressed plants [69]. Thus, it may be speculated that the low flavonol glycoside content in drought-stressed white bean plants results in no impairment of stomatal closure. Given that the simulated drought approach used in our study is more representative of natural soil water scenarios, the lack of flavonol glycoside accumulation may be a consequence of limited de novo biosynthesis via flavonol synthase and their associated glycosyltransferases and/or their turnover to simpler bioactive compounds, such as kaempferol. In fact, the catabolism of flavonol glycosides to their aglycones via β-glucosidases and α-rhamnosidases occurs in plants [42,70]. 

## 5. Conclusions

**Figure 8:**
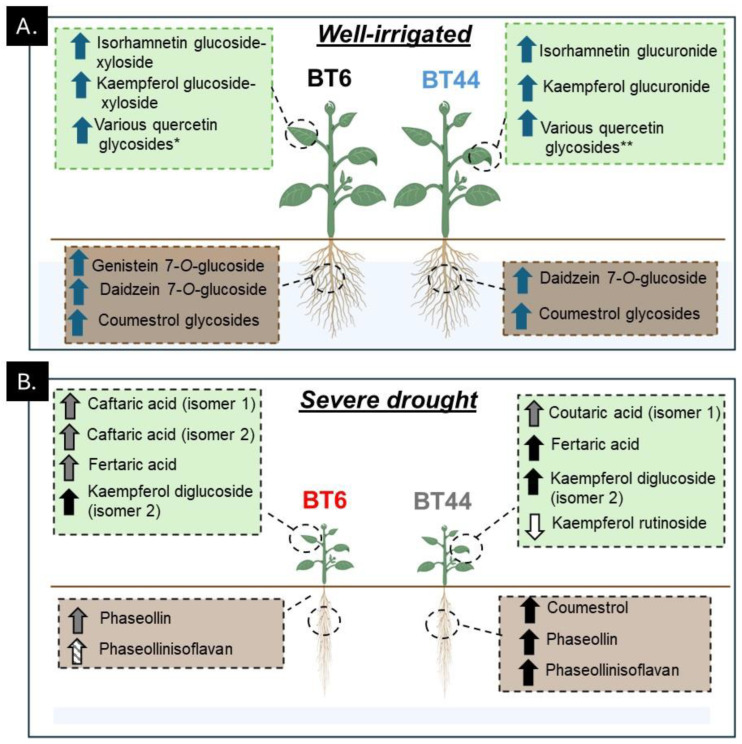
Fine tuning of the phenylpropanoid metabolism in the leaves and roots of two white bean RILs (BT6 and BT44) grown under (**A**) well-irrigated conditions (75% relative soil water content, RSWC) versus (**B**) severe drought (15% RSWC). For each RIL at each RSWC, the green-shaded box and brown-shaded box represent metabolites that were altered over the 12-day period in their leaves and roots, respectively. The arrow next to each metabolite denotes whether a decrease or increase occurred during cultivation. Blue arrow denotes an increase with cultivation under well-irrigated conditions. Grey arrow denotes a transient accumulation of the metabolite with severe drought; black arrow denotes a sustained increase with severe drought; diagonally striped arrow denotes a greater concentration with severe drought relative to well-irrigated plants but there was no alteration over the 12-day time course; white arrow denotes a sustained decrease of the metabolite concentration with severe drought. The single asterisk (*) represents the accumulation of the following quercetin glycosides in BT6 under well-watered conditions: quercetin 3-*O*-glucoside, 3-*O*-galactoside, quercetin xyloside, quercetin dixyloside (isomers 1 and 2), and quercetin glucoside-xyloside (isomers 1 and 2). The double asterisks (**) represent the accumulation of the following quercetin glycosides in BT44 under well-watered conditions: quercetin 3-*O*-glucuronide and quercetin glucuronide-xyloside. Images of plants were created with BioRender.com (accessed on 6 September 2023).

In summary, cultivation of the white bean RILs BT6 and BT44 under 15% RSWC dramatically reduced the FW and DW of their respective roots, stems, and leaves relative to well-watered plants (75% RSWC). The aim of this study was to determine whether the restricted growth attributes of white bean plants under severe drought were associated with alterations in their phenylpropanoid profiles. We found that in both RILs, drought induced the root-specific accumulation of the pterocarpans phaseollin and phaseollinisoflavan, although the timing of these changes varied across the RILs (Figure 8). One major difference between the RILs was the sustained accumulation of coumestrol in BT44 roots, which was not apparent in BT6. Thus, the possibility remains that the gene expression and/or activity of coumestrol and pterocarpan biosynthesis enzymes in the roots of these two RILs are differentially impacted by severe drought. The leaf drought response was highly distinct from that of the roots (Figure 8). The concentrations for the majority of the detected flavonol glycosides were not increased by drought, whereas kaempferol diglucoside (isomer 2) levels were elevated under 15% RSWC. The impact of drought on leaf phenylpropanoid profiles seemed to be more associated with altered phenolic acid composition. In BT44 leaves, drought induced the transient accumulation of coutaric acid (isomer 1) and a sustained increase in the fertaric acid concentration, whereas the concentrations of fertaric acid and both caftaric acid isomers were transiently increased in BT6 leaves (Figure 8). A potential mechanism for the differential accumulation of these phenolic acids across both RILs with drought could be the varied gene expression and/or activity of phenolic acid biosynthesis enzymes such as coumarate 3-hydroxylase and hydroxycinnamoyl-CoA: tartaric acid hydroxycinnamoyl transferase (Figure 1). In summary, previous research revealed that CBB resistance in white bean plants (i.e., BT6) is associated with phytoalexin accumulation in its leaves when challenged with the bacterium that causes the disease [19]. This metabolic response is absent in the CBB-susceptible BT44 [19]. Conversely, some of these same phytoalexins accrued in the roots of both RILs during severe drought, albeit the timing and maintenance of this response varied between BT6 and BT44. Thus, the metabolic response associated with CBB-resistance in BT6 does not strictly predispose this RIL nor does it restrict the CBB-susceptible BT44 from adapting to drought.

## Data Availability

All data are presented within this article and its Supplementary Materials.

## Supplementary Materials

The following supporting information can be downloaded at: https://www.mdpi.com/article/10.3390/metabo14060319/s1, Table S1: UPLC-MS/MS analysis of phenolics, flavonoids, and related metabolites in roots and leaves sampled from white bean recombinant inbred lines BT6 and BT44 during cultivation under varying relative soil water contents; Figure S1: Changes in the total fresh weight of flowers (including undeveloped floral buds) of white bean plants cultivated under 75% relative soil water content (RSWC) and 15% RSWC; Figure S2: Temporal alterations in the concentrations of various leaf isoflavone derivatives during cultivation of white bean RILs BT6 and BT44 under 75% relative soil water content (RSWC) or 15% RSWC; Figure S3: Temporal alterations in the concentrations of various leaf quercetin glycosides during cultivation of white bean RILs BT6 and BT44 under 75% relative soil water content (RSWC) or 15% RSWC.
