# Supplementary material for: Phenylpropanoid Metabolism in Phaseolus vulgaris during Growth under Severe Drought
Source: Metabolites. 2024 May 31;14(6):319. doi: 10.3390/metabo14060319 (PMC11205357; doi:10.3390/metabo14060319)
Supplement: Supplementary file 1 [file metabolites-14-00319-s001.zip › metabolites-3021081-supplementary.pdf]

metabolites

MDPI

Supplementary Materials: Phenylpropanoid Metabolism in *Phaseolus vulgaris* during Growth under Severe Drought

Luis Eduardo Peña Barrena [1], Lili Mats [2], Hugh J. Earl [1] and Gale G. Bozzo [1,*]

[1] Department of Plant Agriculture, University of Guelph, 50 Stone Road East, Guelph, ON N1G 2W1, Canada; lpenabar@uoguelph.ca (L.E.P.B.); hjearl@uoguelph.ca (H.J.E.)
[2] Guelph Research and Development Centre, Agriculture and Agri-Food Canada, 93 Stone Road West, Guelph, ON N1G 5C9, Canada; lili.mats@canada.ca
* Correspondence: gbozzo@uoguelph.ca; Tel.: +1-519-824-4120 (ext. 53036)

Table S1. UPLC-MS/MS analysis of phenolics, flavonoids and related metabolites in roots and leaves sampled from white bean recombinant inbred lines BT6 and BT44 during cultivation under varying relative soil water contents.

Compound	Chemical Formula	Retention Time (min)	Parent Ion [M-H]⁻	Fragment Ion(s) [M-H]⁻
Caftaric acid (isomer 1) [a]	$C_{13}H_{12}O_9$	7.6	311.0410	179.0354, 149.0096, 135.0456, 87.0090
Caftaric acid (isomer 2) [a]	$C_{13}H_{12}O_9$	8.4	311.0410	179.0354, 149.0096, 135.0456, 87.0089
Coutaric acid (isomer 1) [a]	$C_{13}H_{12}O_8$	10.4	295.0461	163.0406, 149.0097, 119.0506, 87.0090
Coutaric acid (isomer 2) [a]	$C_{13}H_{12}O_8$	10.9	295.0461	163.0406, 149.0096, 119.0506, 87.0090
Fertaric acid [a]	$C_{14}H_{14}O_9$	13.0	325.0568	193.0510, 149.0612, 134.0378, 87.0090
p-Coumaric acid [a,b]	$C_9H_8O_3$	17.6	163.0401	163.0402, 119.0501
Coumestrol diglucoside (formic acid adduct) [a]	$C_{27}H_{28}O_{15}$	19.3	637.1419 [c]	429.0829, 266.0221
Daidzein 7-*O*-glucoside [b]	$C_{21}H_{20}O_9$	20.0	415.1037	415.1024, 253.0508, 252.0430
Genistein glucoside (isomer 1) [a]	$C_{21}H_{20}O_{10}$	20.4	431.0986	431.0985, 269.0455, 268.0377
Quercetin rutinoside-xyloside (isomer 1)	$C_{32}H_{38}O_{20}$	22.4	741.1891	301.0358, 300.0278, 178.9991
Quercetin glucoside-xyloside (isomer 1)	$C_{26}H_{28}O_{16}$	22.6	595.1309	595.1312, 301.0356, 300.0279
Quercetin glucuronide-xyloside	$C_{26}H_{26}O_{17}$	22.7	609.1102	301.0355, 300.0261, 178.9992
Quercetin glucoside-xyloside (isomer 2)	$C_{26}H_{28}O_{16}$	22.8	595.1309	595.1312, 463.0898, 301.0357, 300.0280
Daidzein glucoside isomer [a]	$C_{21}H_{20}O_9$	22.8	415.1036	415.1036, 253.0508, 252.0430
Genistein 7-*O*-glucoside [a,b]	$C_{21}H_{20}O_{10}$	22.9	431.0981	431.0984, 269.0457, 268.0378
Kaempferol diglucoside (isomer 1)	$C_{27}H_{30}O_{16}$	23.1	609.1491	609.1492, 285.0414, 284.0327
Kaempferol diglucoside (isomer 2)	$C_{27}H_{30}O_{16}$	23.3	609.1489	609.1492, 285.0414, 284.0327

Quercetin 3-*O*-galactoside [b]	$C_{21}H_{20}O_{12}$	24.5	463.0890	463.0887, 301.0357, 300.0279, 255.0297
Kaempferol rutinoside-xylo-side	$C_{32}H_{38}O_{19}$	24.6	725.1964	725.1967, 285.0410, 284.0329
Quercetin dixyloside (isomer 1)	$C_{25}H_{26}O_{15}$	24.6	565.1214	565.1202, 301.0356, 300.0279
Quercetin 3-*O*-glucuronide [b]	$C_{21}H_{18}O_{13}$	24.8	477.0676	301.0357, 178.9991, 151.0042
Quercetin 3-*O*-glucoside [b]	$C_{21}H_{20}O_{12}$	24.8	463.0887	463.0883, 301.0357, 300.0279, 255.0299
Quercetin 3-*O*-rutinoside [b]	$C_{27}H_{30}O_{16}$	24.9	609.1486	609.1483, 301.0357, 300.0279, 178.9993
Quercetin dixyloside (isomer 2)	$C_{25}H_{26}O_{15}$	25.0	565.1213	565.1225, 301.0349, 300.0278
Kaempferol glucoside-xylo-side	$C_{26}H_{28}O_{15}$	25.1	579.1370	579.1412, 447.1023, 285.0408, 284.0329
Genistein isomer [a]	$C_{15}H_{10}O_5$	25.2	269.0456	269.0455, 225.0560, 147.0092
Isorhamnetin glucoside-xylo-side	$C_{27}H_{30}O_{16}$	25.4	609.1489	315.0520, 314.0434, 300.0280, 299.0198
Genistein glucoside isomer [a]	$C_{21}H_{20}O_{10}$	25.4	431.0986	431.0985, 269.0453, 268.0377
Quercetin xyloside	$C_{20}H_{18}O_{11}$	25.5	433.0779	433.0782, 301.0357, 300.0279, 271.0251
Coumestrol glucoside (isomer 1) [a]	$C_{21}H_{18}O_{10}$	26.1	429.0829	266.0220
Coumestrol glucoside (isomer 2) [a]	$C_{21}H_{18}O_{10}$	26.4	429.0829	429.0828, 267.0291, 266.0220
Kaempferol glucuronide	$C_{21}H_{18}O_{12}$	27.2	461.0730	285.0406, 113.0249, 85.0296
Kaempferol rutinoside	$C_{27}H_{30}O_{15}$	27.3	593.1520	593.1514, 285.0407, 284.0322
Isorhamnetin glucuronide	$C_{22}H_{20}O_{13}$	28.0	491.0833	315.0513, 300.0279, 113.0248, 85.0296
Daidzein [b]	$C_{15}H_{10}O_4$	28.4	253.0510	253.0510, 209.0612
Genistein [b]	$C_{15}H_{10}O_5$	31.2	269.0458	269.0457, 133.0297
Coumestrol [b]	$C_{15}H_8O_5$	33.4	267.0300	267.0301
Kievitone [a]	$C_{20}H_{20}O_6$	37.6	355.1187	355.1188, 193.0871, 161.0247
Phaseollinisoflavan	$C_{20}H_{20}O_4$	38.0	323.1293	323.1292, 282.9800, 201.0920, 135.0452
Phaseollin [a]	$C_{20}H_{18}O_4$	39.2	321.1132	321.1135, 306.0901, 175.0772

[a] Metabolite was not described in the previous study on the metabolic response of white bean leaves to common bacterial blight (Cox et al., 2021). [b] RT and fragmentation pattern match those of an authentic standard. For all other metabolites, their MS parent and fragment ions were compared to an in-house MS/MS library for phenolic acids, flavonol glycosides, isoflavones and their derivatives or those described in the scientific literature as described in the Materials and Methods (Section 2.2). [c] $[M+formic acid-H]^-$ is the major ion, $[M-H]^-$ (m/z = 591.1355) was not detected in most samples

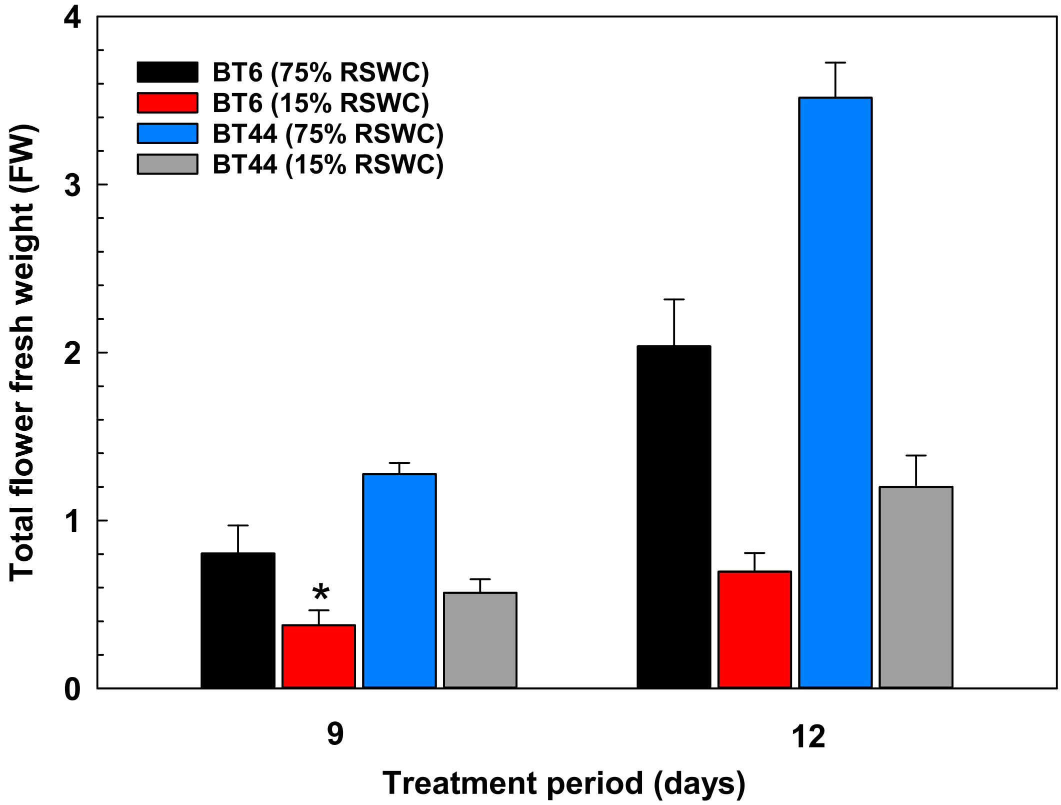

Figure S1. Changes in the total fresh weight of flowers (including undeveloped floral buds) of white bean plants cultivated under 75% relative soil water content (RSWC) and 15% RSWC. The floral organs were harvested at the time of their initial appearance within each RIL at each RSWC which was on the 9th day of the varying RSWC time course (day 23 post-emergence) and again on day 12 of this experiment (day 26 post-emergence). Each datum within a plot represents the mean ± standard error four experimental replicates with exception of * of the BT6 plants sampled on day 9 of the 15% RSWC, where only three of the four replicates were assessed.

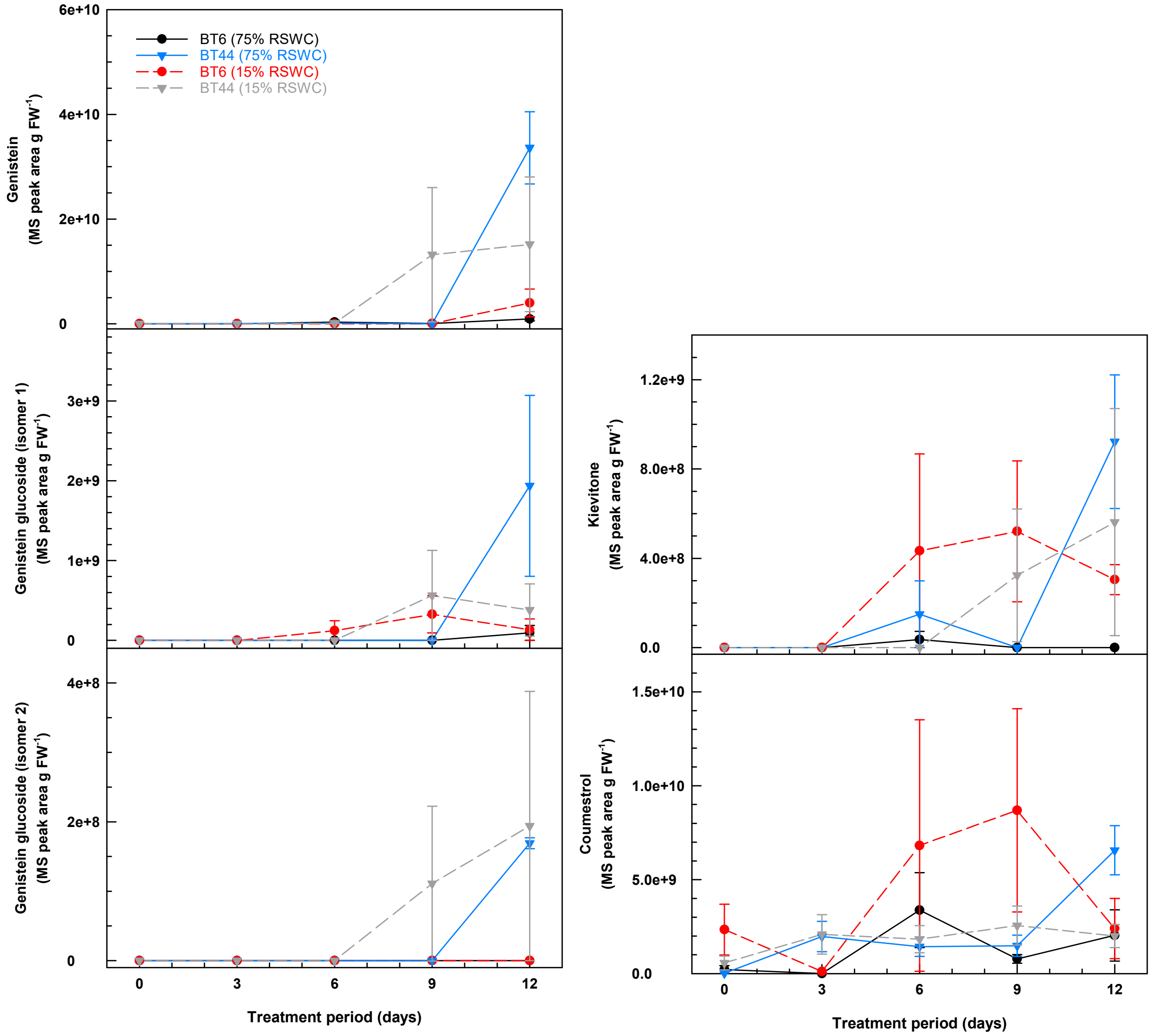

Figure S2. Temporal alterations in the concentrations of various leaf isoflavone derivatives during cultivation of white bean RILs BT6 and BT44 under 75% relative soil water content (RSWC) or 15% RSWC. Each metabolite concentration is based on the MS peak area associated with the intensity of the metabolite's parent ion (m/z) divided by the precise milligrams of tissue extracted and then extrapolated and expressed as MS peak area g FW^{-1}. Each datum within a panel represents the mean ± standard error of four plants with a plant sampled from each of four experimental block replicates.

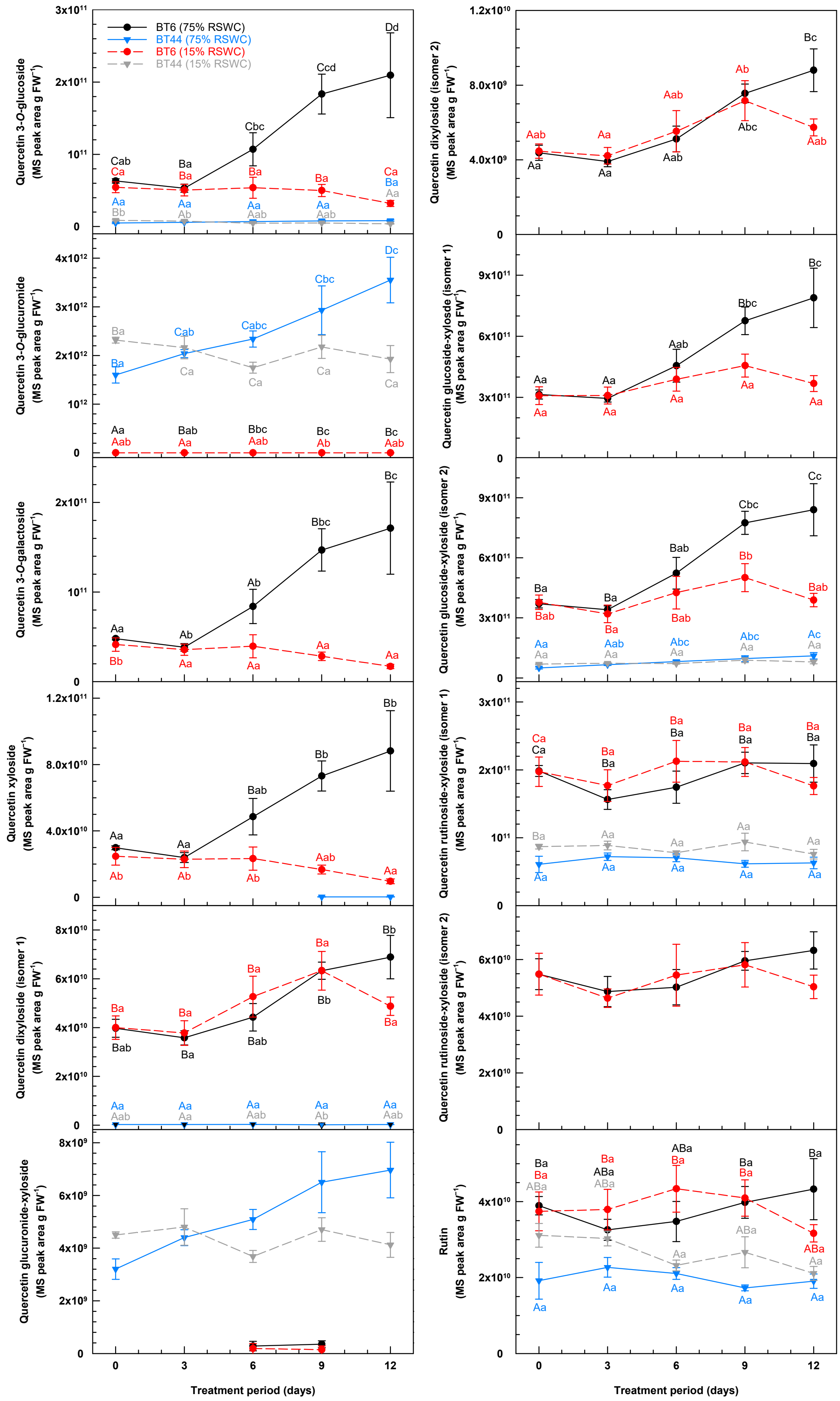

Figure S3. Temporal alterations in the concentrations of various leaf quercetin glycosides during cultivation of white bean RILs BT6 and BT44 under 75% relative soil water content (RSWC) or 15% RSWC. Each metabolite concentration is based on the MS peak area associated with the intensity of the metabolite's parent ion (m/z) divided by the precise milligrams of tissue extracted and then extrapolated and expressed as MS peak area g FW^{-1}. Each datum within a panel represents the mean

± standard error of four plants with a plant sampled from each of four experimental block replicates. Means sharing the same letter are not statistically different. Upper case letters indicate significant differences across the RILs and their treatments within a single sampling day. Lower case letters indicate significant differences within a treatment across the 12-day treatment period. Black and blue lettering correspond to the proximal BT6 and BT44 75% RSWC treatment data, respectively; red and grey lettering correspond to the proximal BT6 and BT44 15% RSWC treatment data, respectively. For the quercetin glucuronide-xyloside and quercetin rutinoside-xyloside (isomer 2) panels, respective BT44 and BT6 data are not accompanied by statistical letters as there was no significant difference across any of the compared means.
